# Th17/Treg imbalance in COPD progression: A temporal analysis using a CS-induced model

**DOI:** 10.1371/journal.pone.0209351

**Published:** 2019-01-10

**Authors:** Juliana Tiyaki Ito, Daniela Aparecida de Brito Cervilha, Juliana Dias Lourenço, Natália Gomes Gonçalves, Rildo Aparecido Volpini, Elia Garcia Caldini, Gilles Landman, Chin Jia Lin, Ana Paula Pereira Velosa, Walcy Paganelli Rosolia Teodoro, Iolanda de Fátima Lopes Calvo Tibério, Thais Mauad, Milton de Arruda Martins, Mariangela Macchione, Fernanda Degobbi Tenorio Quirino dos Santos Lopes

**Affiliations:** 1 Department of Clinical Medicine, Laboratory of Experimental Therapeutics, School of Medicine, University of Sao Paulo, Sao Paulo, Brazil; 2 Department of Pathology, Laboratory of Molecular Pathology, School of Medicine, University of Sao Paulo, Sao Paulo, Brazil; 3 Department of Clinical Medicine, Basic Research Laboratory on Kidney Diseases, School of Medicine, University of Sao Paulo, Sao Paulo, Brazil; 4 Department of Pathology, Laboratory of Cell Biology, School of Medicine, University of Sao Paulo, Sao Paulo, Brazil; 5 Department of Pathology, Multi-purpose Laboratory of Molecular Pathology, Federal University of São Paulo, São Paulo, Brazil; 6 Department of Clinical Medicine, Laboratory of Extracellular Matrix, School of Medicine, University of Sao Paulo, Sao Paulo, Brazil; 7 Department of Pathology, Experimental Air Pollution Laboratory, School of Medicine, University of Sao Paulo, Sao Paulo, Brazil; University of Pittsburgh, UNITED STATES

## Abstract

**Background:**

The imbalance between pro- and anti-inflammatory immune responses plays a pivotal role in chronic obstructive pulmonary disease (COPD) development and progression. To clarify the pathophysiological mechanisms of this disease, we performed a temporal analysis of immune response-mediated inflammatory progression in a cigarette smoke (CS)-induced mouse model with a focus on the balance between Th17 and Treg responses.

**Methods:**

C57BL/6 mice were exposed to CS for 1, 3 or 6 months to induce COPD, and the control groups were maintained under filtered air conditions for the same time intervals. We then performed functional (respiratory mechanics) and structural (alveolar enlargement) analyses. We also quantified the NF-κB, TNF-α, CD4, CD8, CD20, IL-17, IL-6, FOXP3, IL-10, or TGF-β positive cells in peribronchovascular areas and assessed FOXP3 and IL-10 expression through double-label immunofluorescence. Additionally, we evaluated the gene expression of NF-κB and TNF in bronchiolar epithelial cells.

**Results:**

Our CS-induced COPD model exhibited an increased proinflammatory immune response (increased expression of the NF-κB, TNF-α, CD4, CD8, CD20, IL-17, and IL-6 markers) with a concomitantly decreased anti-inflammatory immune response (FOXP3, IL-10, and TGF-β markers) compared with the control mice. These changes in the immune responses were associated with increased alveolar enlargement and impaired lung function starting on the first month and third month of CS exposure, respectively, compared with the control mice.

**Conclusion:**

Our results showed that the microenvironmental stimuli produced by the release of cytokines during COPD progression lead to a Th17/Treg imbalance.

## Introduction

Chronic obstructive pulmonary disease (COPD) is characterized by a progressive airflow limitation that is not fully reversible and is associated with a chronic inflammatory response in the lungs [1]. COPD is one of the main causes of morbidity worldwide and is estimated to become the third most common cause of death by 2030 [2].

Smoking has been confirmed to be the major risk factor for COPD development. However, only 15–20% of smokers develop this disease, which suggests that individual intrinsic factors are responsible for COPD progression [3–5].

Several studies have highlighted the importance of innate [6, 7] and adaptive [8, 9] immune responses in the pathophysiology of COPD. Moreover, the imbalance between proinflammatory and anti-inflammatory immune responses mediated by the different subsets of T helper (Th) cells, such as Th17 and regulatory T (Treg) cells, respectively, plays a pivotal role in the progression of this disease [10, 11].

The Th17 response has strong proinflammatory abilities mediated by the release of interleukin (IL)-17 [12], which can facilitate the proliferation of T cells and the expression of various inflammatory mediators [13]. In contrast, Treg cells are responsible for the secretion of anti-inflammatory cytokines, such as IL-10 and TGF-β, which promote the control of the inflammatory response in COPD [11, 14–17]. Additionally, the abnormal Treg response observed in COPD patients might lead to persistent inflammation and thus progression of the disease [17, 18].

Th17 differentiation is positively regulated by IL-6, TGF-β, and IL-1β but negatively regulated by IL-10 [11]. The differentiation of Tregs is also induced by TGF-β but can be inhibited by IL-6 [19].

Despite the Th17/Treg imbalance and the worsening of alveolar enlargement and lung function observed in clinical and experimental COPD studies [10, 11, 15, 16], the pathophysiological mechanisms at different time points can only be evaluated using animal models, and this information is crucial for obtaining an improved understanding of the changes between the pro- and anti-inflammatory immune responses involved in the development and progression of COPD.

In this study, we used a CS-induced animal model and performed a temporal analysis of the inflammatory progression mediated by the adaptive immune response with a focus on the Th17/Treg responses.

## Materials and methods

### Experimental groups

Male C57BL/6 mice (aged 6–8 weeks and weighing 20–25g) were randomly divided into groups exposed to CS for 1, 3 or 6 months, and the mice in the control groups were maintained under filtered air conditions for the same time intervals (1, 3 or 6 months). All the animals received human care in compliance with the Guide for the Care and Use of Laboratory Animals published by the US National Institutes of Health (NIH Publication N°. 85–23, revised 1996). Our protocol was approved by the ethical committee of the School of Medicine of the University of São Paulo—protocol number 076/14 (São Paulo, Brazil).

### CS exposure protocol

The animals were exposed to CS as previously described by Toledo et al. [20]. The flow rate was set such that the carbon monoxide (CO) levels ranged from 250 to 350 parts per million (ppm). Approximately 12 ± 1 commercially filtered cigarettes were used per exposure (0.8 mg of nicotine, 10 mg of tar, and 10 mg of CO per cigarette), resulting in a total particulate matter concentration of 354.8 ± 50.3 μg/m^3^/day. The animals were maintained in the CS environment for 30 min for each exposure, and the exposures were repeated twice per day, 5 days per week for a period of 1, 3 or 6 months. The control groups were maintained under filtered air conditions.

### Evaluation of respiratory mechanics

Twenty-four hours after the end of the exposure period, the mice were intraperitoneally anesthetized with thiopental (50 mg/kg), tracheostomized, and mechanically ventilated using a FlexiVent ventilator (Scireq, Montreal, QC, Canada). Using the forced oscillatory technique and a constant phase model [21], values for the parameters airway resistance (Raw), tissue damping (Gtis), and tissue elastance (Htis) were obtained and normalized to the body weight [22] because the mice exposed to CS exhibited a lower body mass at the end of the exposure periods compared with the mice in the respective control groups (1^st^ month: P < 0.0001, 21.71 ± 1.62 vs. 25.05 ± 1.39; 3^rd^ month: P = 0.0005, 24.19 ± 0.79 vs. 27.60 ± 1.74; and 6^th^ month: P < 0.0001, 26.64 ± 2.92 vs. 34.31 ± 2.89).

### Lung preparation

After assessment of the respiratory mechanics, the mice were euthanized by abdominal aortic exsanguination, and the lungs were removed and fixed at a constant pressure (20 cmH_2_O) using 10% buffered formalin infused through the trachea for 24 h. The lungs were subsequently embedded in paraffin and cut into 5-μm-thick sections.

#### Immunohistochemistry

Lung tissue sections were immunostained with the following primary antibodies: rabbit polyclonal anti-nuclear factor-*kappa* B (NF-κB) p65 (sc-109, 1:100, Santa Cruz Biotechnology, CA, USA), mouse anti-tumor necrosis factor-*alpha* (TNF-α) (sc-52746, 1:5000, Santa Cruz Biotechnology, CA, USA), rat monoclonal anti-CD4 (sc-13573, 1:10, Santa Cruz Biotechnology, CA, USA), rabbit polyclonal anti-CD8 (ab4055, 1:200, Abcam, Cambridge, UK), rabbit polyclonal anti-CD20 (orb10307, 1:200, Biorbyt LLC, CA, USA), rabbit polyclonal anti-IL-17 (ab79056, 1:200, Abcam, Cambridge, UK), goat polyclonal anti-IL-6 (sc-1265, 1:400, Santa Cruz Biotechnology, CA, USA), rabbit polyclonal anti-forkhead box P3 (FOXP3) (ab54501, 1:700, Abcam, Cambridge, UK), rat monoclonal anti-IL-10 (sc-73309, 1:100, Santa Cruz Biotechnology, CA, USA), and rabbit polyclonal anti-TGF-β (sc-146, 1:300, Santa Cruz Biotechnology, CA, USA). Species-specific secondary antibodies in conjunction with a Vector ABC kit (Vector Laboratories, CA, USA) or Histofine Simple Stain MAX PO anti-rabbit polymer (Nichirei Biosciences, Tokyo, JP) were used for the reactions. All the sections were stained using the chromogen 3,3’-diaminobenzidine (DAB, Sigma-Aldrich, MO, USA) and were counterstained with Harris’s hematoxylin (Merck, Darmstadt, Germany). Samples that were not incubated with the primary antibody were used as a negative controls, and bovine serum albumin (BSA) was used on the tissue samples.

#### Immunofluorescence

The lung specimens were incubated with rabbit polyclonal anti-FOXP3 (sc-28705, 1:200, Santa Cruz Biotechnology, CA, USA) and goat polyclonal anti-IL-10 (sc-1783, 1:1200, Santa Cruz Biotechnology, CA, USA) and then with Alexa 488-conjugated goat anti-rabbit IgG (1:150, Invitrogen, Eugene, OR, USA) and Alexa 546-conjugated donkey anti-goat IgG (1:150, Invitrogen, Eugene, OR, USA) at room temperature. The nuclei were then counterstained with DAPI (Molecular Probes, Invitrogen, Eugene, OR, USA). Lung images were captured using a fluorescence microscope (OLYMPUS BX51) at 400× magnification, and serial images were overlaid using ImageJ software (US National Institutes of Health).

#### Morphometry

Lung tissue sections were stained with hematoxylin and eosin (H&E) to evaluate the mean linear intercept (Lm), which serves as an indicator of the mean diameter of airspaces, as described previously [23] using an eyepiece with a known area attached to the microscope ocular lens [24]. For each animal, 20 nonoverlapping fields around the airways and in the distal lung parenchyma were assessed at 400× magnification, and the Lm values are presented in micrometers (μm).

Immunohistochemically stained lung tissue sections were scanned using a high-resolution digital scanner (Pannoramic SCAN, 3DHISTECH Ltd., Budapest, HU), and 5 to 10 peribronchovascular areas [25] at 200× magnification were analyzed for each animal. All the inflammatory mediators were measured with imaging analysis software (Pannoramic Viewer 1.15.4, 3DHISTECH Ltd., Budapest, HU) by counting the number of positive cells and dividing by the respective peribronchovascular total area calculated by the software (results are expressed as cells/μm^2^).

### Tissue preparation for laser capture microdissection (LCM)

For real-time RT-PCR analysis, the lungs were removed *en bloc*, inflated with diluted Tissue-Tek OCT (Sakura Finetek USA., Torrance, CA, USA) (50% vol/vol) in ribonuclease (RNase)-free phosphate-buffered saline with 10% sucrose, immediately frozen in liquid nitrogen, and stored at -80°C [26]. The lungs were sectioned (12 μm) with a cryostat (Leica CM3050 S, Leica Biosystems, Wetzlar, DEU) and stained with 0.5% Nissl (Cresyl violet acetate) (Sigma Chemical, St. Louis, MO, USA)/0.1 M sodium acetate buffer for laser capture microdissection (LCM) of the bronchiolar epithelial cells using a Zeiss PALM Microbeam Laser Microdissection System (Carl Zeiss Microscopy GmbH, Jena, DEU). A total of 20,000 to 40,000 bursts of the laser were used to collect a minimum of 5,000 cells from each animal.

#### RNA extraction and reverse transcription

Total RNA from the laser-captured bronchiolar epithelial cells was extracted using an RNAqueous-Micro Total RNA Isolation kit (Ambion, Applied Biosystems, Foster City, CA, USA) following the manufacturer’s instructions. The quantity and quality of the RNA extract were assessed by microcapillary electrophoresis with a Bioanalyzer 2100 (Agilent Technologies, Santa Clara, CA, USA) using an Agilent RNA 6000 Pico kit (Agilent Technologies, Santa Clara, CA, USA). Total RNA was reverse transcribed for complementary DNA synthesis (cDNA) using a High-Capacity RNA-to-cDNA kit (Applied Biosystems, Foster City, CA, USA) following the manufacturer’s instructions.

#### Real-time RT-PCR

The samples from each animal were assayed in duplicate, and real-time RT-PCR was performed with a StepOnePlus Real-Time PCR System (Applied Biosystems, Foster City, CA, USA). The polymerase chain reactions were performed with 1 μL of target TaqMan Gene Expression (NF-κB–Mm00476361_m1, TNF-α–Mm00443258_m1), 10 μL of TaqMan Universal Master Mix II (Applied Biosystems, Foster City, CA, USA), 7 μL of DEPC-treated water (Ambion, Applied Biosystems, Foster City, CA, USA), and 2 μL of cDNA. The relative expression levels of the target gene were quantified using the 2^-ΔΔCt^ approach [27] with the housekeeping β-actin (Mm01205647_g1) as the endogenous control gene.

### Statistical analysis

The statistical analyses were performed using GraphPad Prism 5.0 (GraphPad, San Diego, CA, USA). The normality of the data distribution was verified with the Shapiro-Wilk test, and the data are presented as the means ± standard deviations (SDs). Firstly, we compared the Smoke groups and their respective Control groups at different time points using t-test or Mann-Whitney test, depending on the normality of the data.

Because we did not observe significant differences among the Control groups at different time points (Table 1), we performed a temporal analysis of the three Smoke groups. For these comparisons, we used one-way analysis of variance (ANOVA) followed by Tukey’s post-test and Kruskal-Wallis test followed by Dunn’s post-test for parametric and non-parametric data, respectively. The differences were considered significant at P < 0.05.

**Table 1:** Comparisons among Control groups at different time points.

Control groups					
	1^st^ month	3^rd^ month	6^th^ month	P	Statistical test
**Raw (cmH**_**2**_**O.s.kg/mL)**	0.2446 ± 0.0434	0.2362 ± 0.0089	0.2091 ± 0.0359	0.3053	One-way ANOVA and Tukey’s post-test
**Gtis (cmH**_**2**_**0.s**^**(1-a)**^**.kg/mL)**	0.1630 ± 0.0145	0.1857 ± 0.0326	0.1751 ± 0.0340	0.3116	One-way ANOVA and Tukey’s post-test
**Htis (cmH**_**2**_**0.s**^**(1-a)**^**.kg/mL)**	0.8493 ± 0.0908	0.9775 ± 0.2279	0.9364 ± 0.2270	0.3690	One-way ANOVA and Tukey’s post-test
**Lm–peribronchial area (μm)**	42.97 ± 1.82	40.80 ± 0.7380	43.15 ± 2.42	0.1004	One-way ANOVA and Tukey’s post-test
**Lm–distal parenchyma (μm)**	42.43 ± 2.82	41.15 ± 1.86	44.36 ± 2.53	0.1721	Kruskal-Wallis test and Dunn’s post-test
**CD4**^**+**^ **T cells (× 10**^**−4**^ **cells/μm**^**2**^**)**	0.3483 ± 0.1030	0.3401 ± 0.2909	0.2791 ± 0.2944	0.3321	Kruskal-Wallis test and Dunn’s post-test
**CD8**^**+**^ **T cells (× 10**^**−4**^ **cells/μm**^**2**^**)**	0.1949 ± 0.1395	0.3213 ± 0.1780	0.1872 ± 0.1511	0.2689	One-way ANOVA and Tukey’s post-test
**CD20**^**+**^ **B cells (× 10**^**−4**^ **cells/μm**^**2**^**)**	0.2649 ± 0.2496	0.2253 ± 0.1728	0.4942 ± 0.1410	0.0959	One-way ANOVA and Tukey’s post-test
**IL-17**^**+**^ **cells (× 10**^**−4**^ **cells/μm**^**2**^**)**	0.1627 ± 0.1068	0.1462 ± 0.0798	0.2562 ± 0.1500	0.2380	One-way ANOVA and Tukey’s post-test
**IL-6**^**+**^ **cells (× 10**^**−4**^ **cells/μm**^**2**^**)**	0.2828 ± 0.1631	0.3159 ± 0.0992	0.2623 ± 0.1347	0.8397	One-way ANOVA and Tukey’s post-test
**Treg cells (× 10**^**−4**^ **cells/μm**^**2**^**)**	0.9283 ± 0.4213	1.1170 ± 0.1482	0.9438 ± 0.4752	0.6277	Kruskal-Wallis test and Dunn’s post-test
**IL-10**^**+**^ **cells (× 10**^**−4**^ **cells/μm**^**2**^**)**	0.6085 ± 0.2838	0.9203 ± 0.3855	0.9398 ± 0.1774	0.0836	One-way ANOVA and Tukey’s post-test
**TGF-β**^**+**^ **cells (× 10**^**−4**^ **cells/μm**^**2**^**)**	0.4295 ± 0.2263	0.6334 ± 0.2047	0.5069 ± 0.2369	0.5604	One-way ANOVA and Tukey’s post-test

## Results

### Comparisons between the Smoke groups and their respective Control groups

#### Respiratory mechanics and lung morphometry

**Fig 1:**
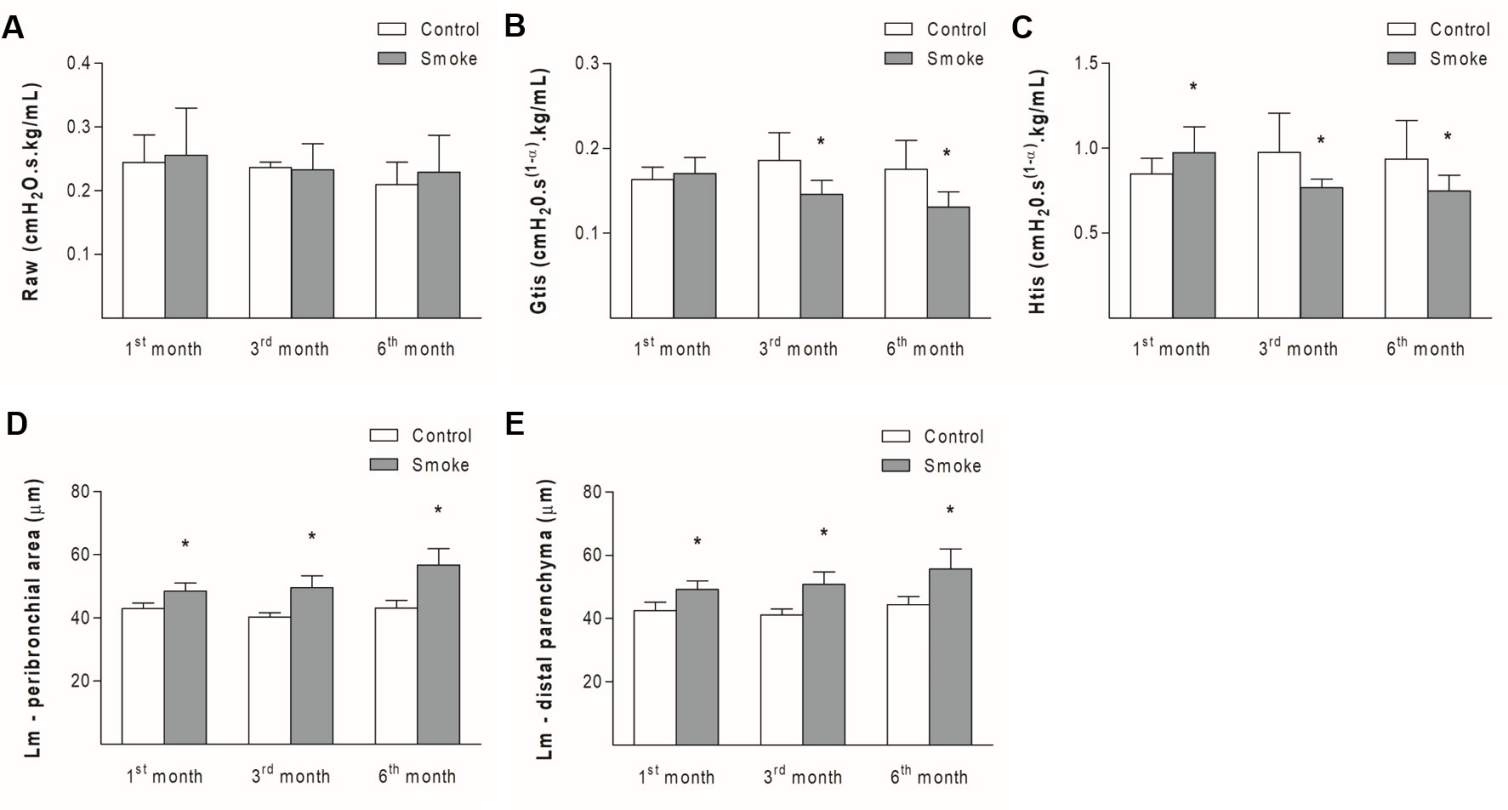
Respiratory mechanics parameters and alveolar enlargement. The values of Raw (A), Gtis (B), Htis (C), Lm of the peribronchial areas (D) and lung distal parenchyma (E) obtained for the Control groups after 1 (n = 10, 10, 10, 7, and 9, respectively), 3 (n = 4, 4, 5, 6, and 5, respectively), and 6 (n = 4, 6, 6, 6, and 7, respectively) months and the Smoke groups after 1 (n = 11, 11, 11, 10, and 11, respectively), 3 (n = 7, 7, 5, 6, and 6, respectively), and 6 (n = 7, 10, 10, 11, and 11, respectively) months are presented as the means ± SDs. (A) No significant difference was detected. (B) Significant differences were observed after 3 (*P = 0.0242, Mann-Whitney test) and 6 (*P = 0.0173, Mann-Whitney test) months. (C) Significant differences were found after 1 (*P = 0.0265, Mann-Whitney test), 3 (*P = 0.0317, Mann-Whitney test), and 6 (*P = 0.0339, t-test) months. (D) Significant differences were found after 1 (*P = 0.0002, Mann-Whitney test), 3 (*P = 0.0002, t-test), and 6 (*P = 0.0001, t-test) months. (E) Significant differences were detected after 1 (*P = 0.0002, Mann-Whitney test), 3 (*P = 0.0008, t-test), and 6 (*P = 0.0004, t-test) months.

Although there were no significant differences in the Raw parameter between the Smoke and Control groups at the different time points (Fig 1A; 1^st^ month, P = 0.6784, t-test; 3^rd^ month, P = 0.7879, Mann-Whitney test; 6^th^ month, P = 0.6485, Mann-Whitney test), decreases in the Gtis value were observed in the Smoke groups after 3 and 6 months of exposure compared with the respective Control groups (Fig 1B; 1^st^ month, P = 0.4597, Mann-Whitney test; 3^rd^ month, P = 0.0242, Mann-Whitney test; 6^th^ month, P = 0.0173, Mann-Whitney test). One month of CS exposure resulted in an increased Htis value, whereas 3 and 6 months of exposure decreased the value of this parameter (Fig 1C; 1^st^ month, P = 0.0265, Mann-Whitney test; 3^rd^ month, P = 0.0317, Mann-Whitney test; 6^th^ month, P = 0.0339, t-test).

One month of CS exposure increased the Lm in the peribronchial area (Fig 1D; 1^st^ month, P = 0.0002, Mann-Whitney test; 3^rd^ month, P = 0.0002, t-test; 6^th^ month, P = 0.0001, t-test) and the distal parenchyma (Fig 1E; 1^st^ month, P = 0.0002, Mann-Whitney test; 3^rd^ month, P = 0.0008, t-test; 6^th^ month, P = 0.0004, t-test), suggesting alveolar enlargement, and these observations were also observed after 3 and 6 months of exposure.

#### Immune response evaluation

**Fig 2:**
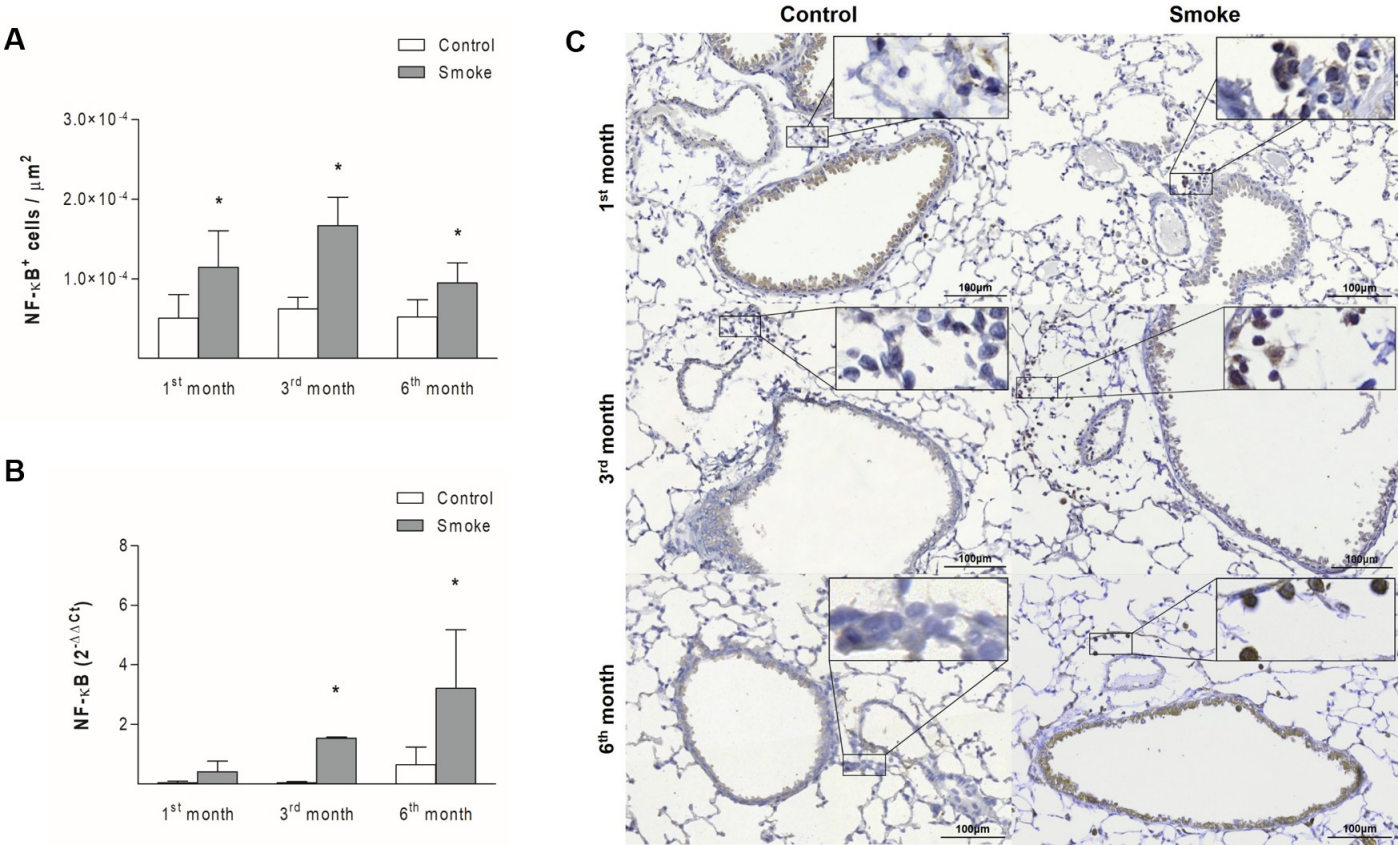
NF-κB expression in peribronchovascular areas and bronchiolar epithelial cells. The numbers of NF-κB^+^ cells in peribronchovascular areas (A) and the NF-κB gene expression levels in bronchiolar epithelial cells (B) obtained for the Control groups after 1 (n = 10 and 5, respectively), 3 (n = 6 and 5, respectively), and 6 (n = 7 and 4, respectively) months and the Smoke groups after 1 (n = 8 and 5, respectively), 3 (n = 5 and 4, respectively), and 6 (n = 10 and 4, respectively) months are presented as the means ± SDs. (A) Significant differences were found after 1 (*P = 0.0025, t-test), 3 (*P = 0.0001, t-test), and 6 (*P = 0.0025, t-test) months. (B) Significant differences were found after 3 (*P = 0.0001, Mann-Whitney test) and 6 (*P = 0.0357, Mann-Whitney test) months. (C) Representative photomicrographs of NF-κB^+^ cells in peribronchovascular areas are shown at 200× magnification, and images at 1000× magnification are shown in each insert.

**Fig 3:**
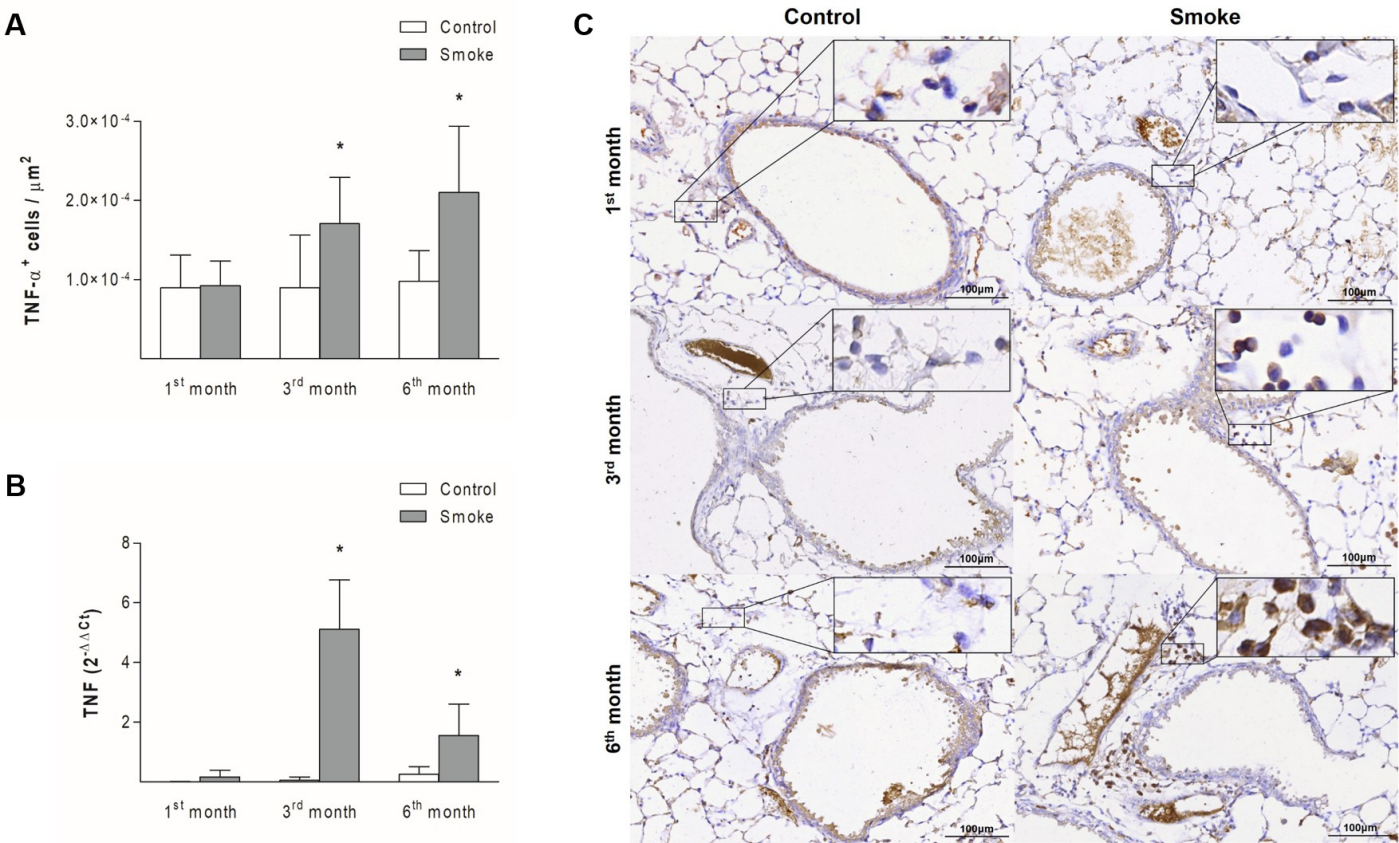
TNF-α expression in peribronchovascular areas and bronchiolar epithelial cells. The numbers of TNF-α^+^ cells in peribronchovascular areas (A) and the TNF gene expression levels in bronchiolar epithelial cells (B) obtained for the Control groups after 1 (n = 10 and 5, respectively), 3 (n = 6 and 5, respectively), and 6 (n = 7 and 4, respectively) months and the Smoke groups after 1 (n = 10 and 5, respectively), 3 (n = 6 and 4, respectively), and 6 (n = 11 and 4, respectively) months are presented as the means ± SDs. (A) Significant differences were found after 3 (*P = 0.0486, t-test) and 6 (*P = 0.0066, Mann-Whitney test) months. (B) Significant differences were found after 3 (*P = 0.0004, t-test) and 6 (*P = 0.0286, Mann-Whitney test) months. (C) Representative photomicrographs of TNF-α^+^ cells in peribronchovascular areas are shown at 200× magnification, and images at 1000× magnification are shown in each insert.

The numbers of NF-κB^+^ cells in peribronchovascular areas were increased starting after one month of CS exposure (Fig 2A; 1^st^ month, P = 0.0025, t-test; 3^rd^ month, P = 0.0001, t-test; 6^th^ month, P = 0.0025, t-test), whereas the gene expression of this mediator in bronchiolar epithelial cells was higher starting after 3 months of exposure (Fig 2B; 1^st^ month, P = 0.0571, Mann-Whitney test; 3^rd^ month, P = 0.0001, Mann-Whitney test; 6^th^ month, P = 0.0357, Mann-Whitney test). The numbers of TNF-α^+^ cells (Fig 3A; 1^st^ month, P = 0.8937, t- test; 3^rd^ month, P = 0.0486, t-test; 6^th^ month, P = 0.0066, Mann-Whitney test) and the level of TNF gene expression (Fig 3B; 1^st^ month, P = 0.0556, Mann-Whitney test; 3^rd^ month, P = 0.0004, t-test; 6^th^ month, P = 0.0286, Mann-Whitney test) were increased starting on the third month of CS exposure in both lung compartments assessed.

**Fig 4:**
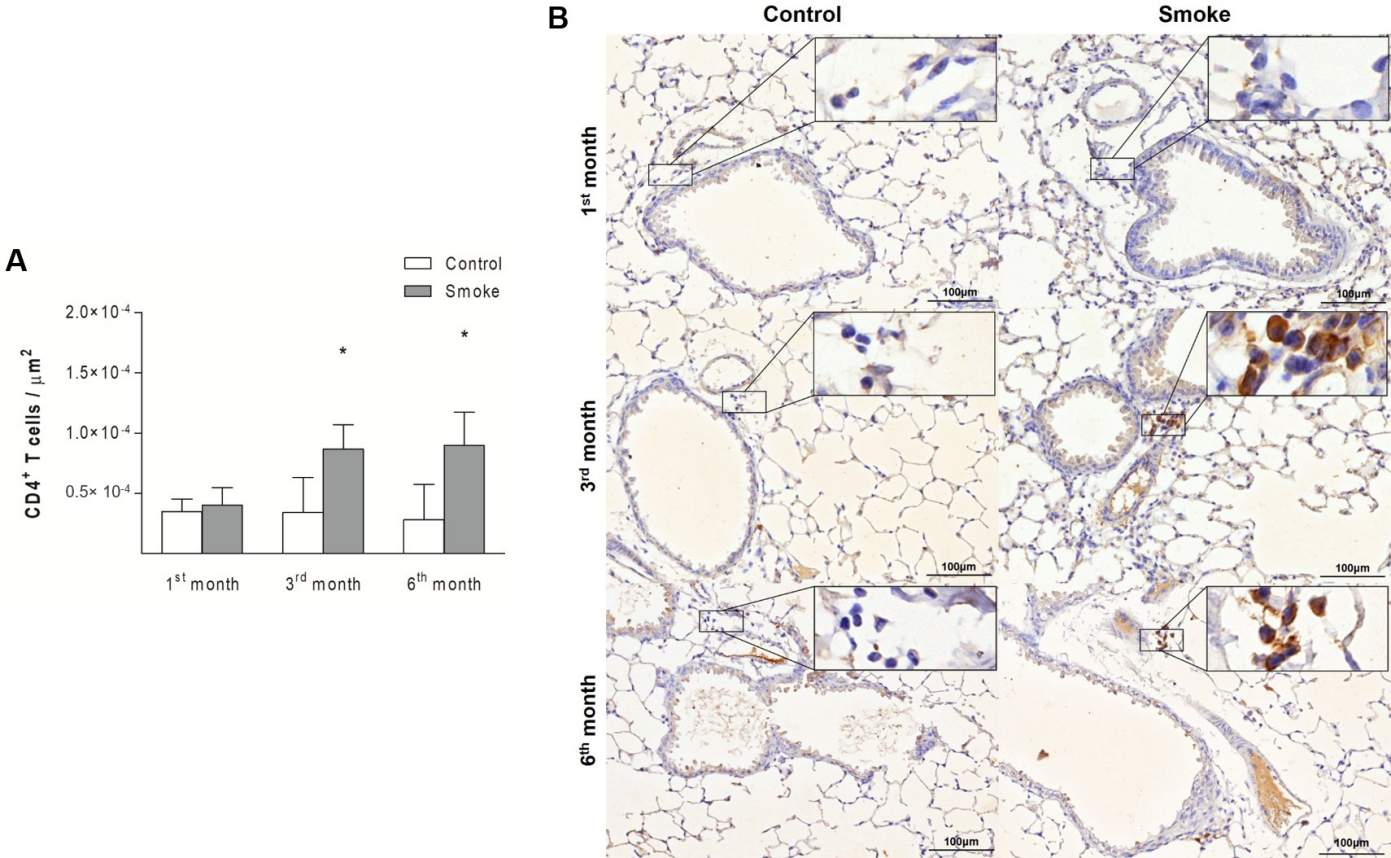
CD4^+^ T cells in peribronchovascular areas. The numbers of CD4^+^ T cells in peribronchovascular areas obtained for the Control groups after 1 (n = 10), 3 (n = 6), and 6 (n = 7) months and the Smoke groups after 1 (n = 9), 3 (n = 5), and 6 (n = 7) months are presented as the means ± SDs. (A) Significant differences were found after 3 (*P = 0.0190, Mann-Whitney test) and 6 (*P = 0.0023, Mann-Whitney test) months. (B) Representative photomicrographs of CD4^+^ T cells in peribronchovascular areas are shown at 200× magnification, and images at 1000× magnification are shown in each insert.

**Fig 5:**
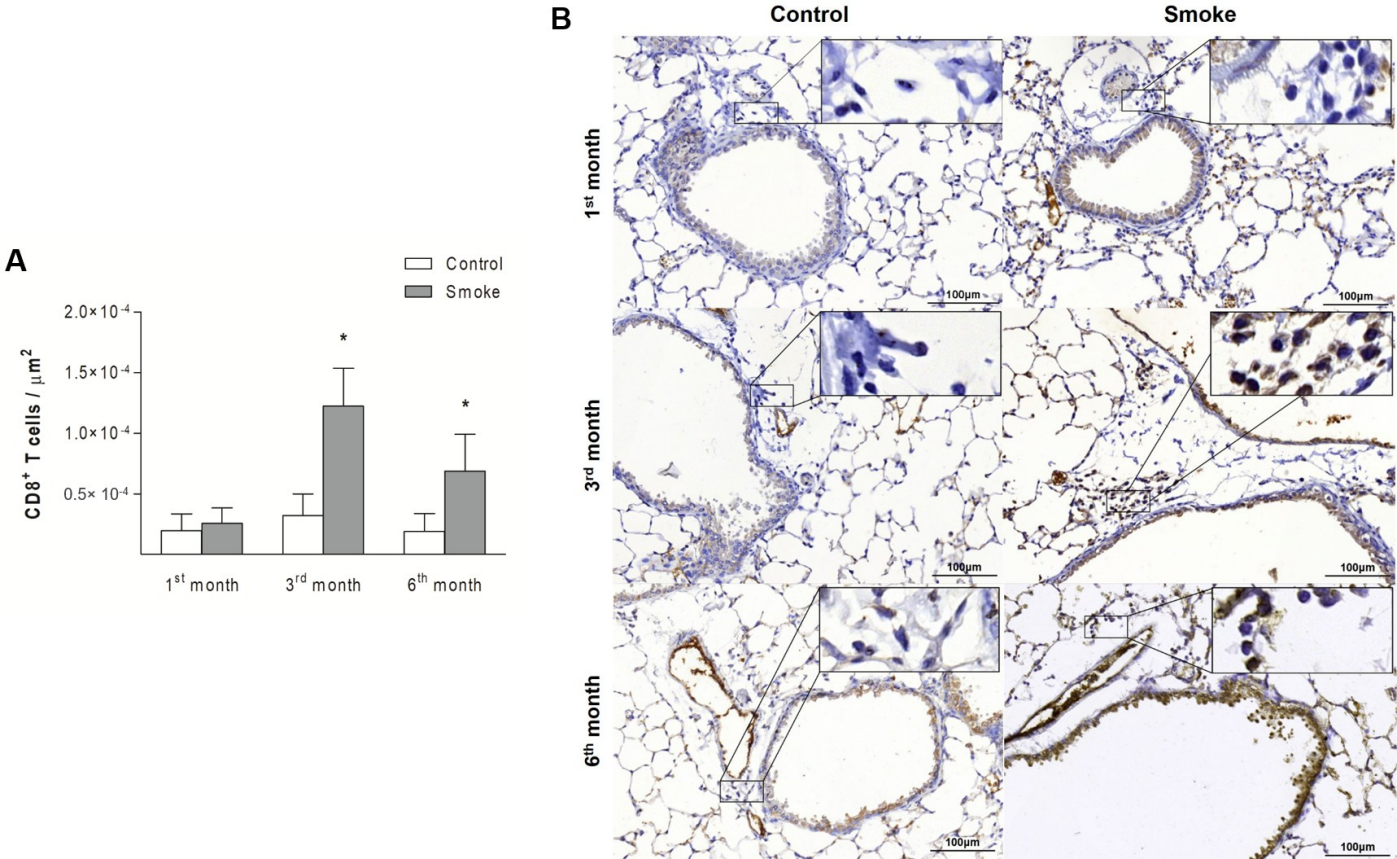
CD8^+^ T cells in peribronchovascular areas. The numbers of CD8^+^ T cells in peribronchovascular areas obtained for the Control groups after 1 (n = 10), 3 (n = 5), and 6 (n = 7) months and the Smoke groups after 1 (n = 10), 3 (n = 7), and 6 (n = 9) months are presented as the means ± SDs. (A) Significant differences were found after 3 (*P = 0.0002, t-test) and 6 (*P = 0.0014, t-test) months. (B) Representative photomicrographs of CD8^+^ T cells in peribronchovascular areas are shown at 200× magnification, and images at 1000× magnification are shown in each insert.

**Fig 6:**
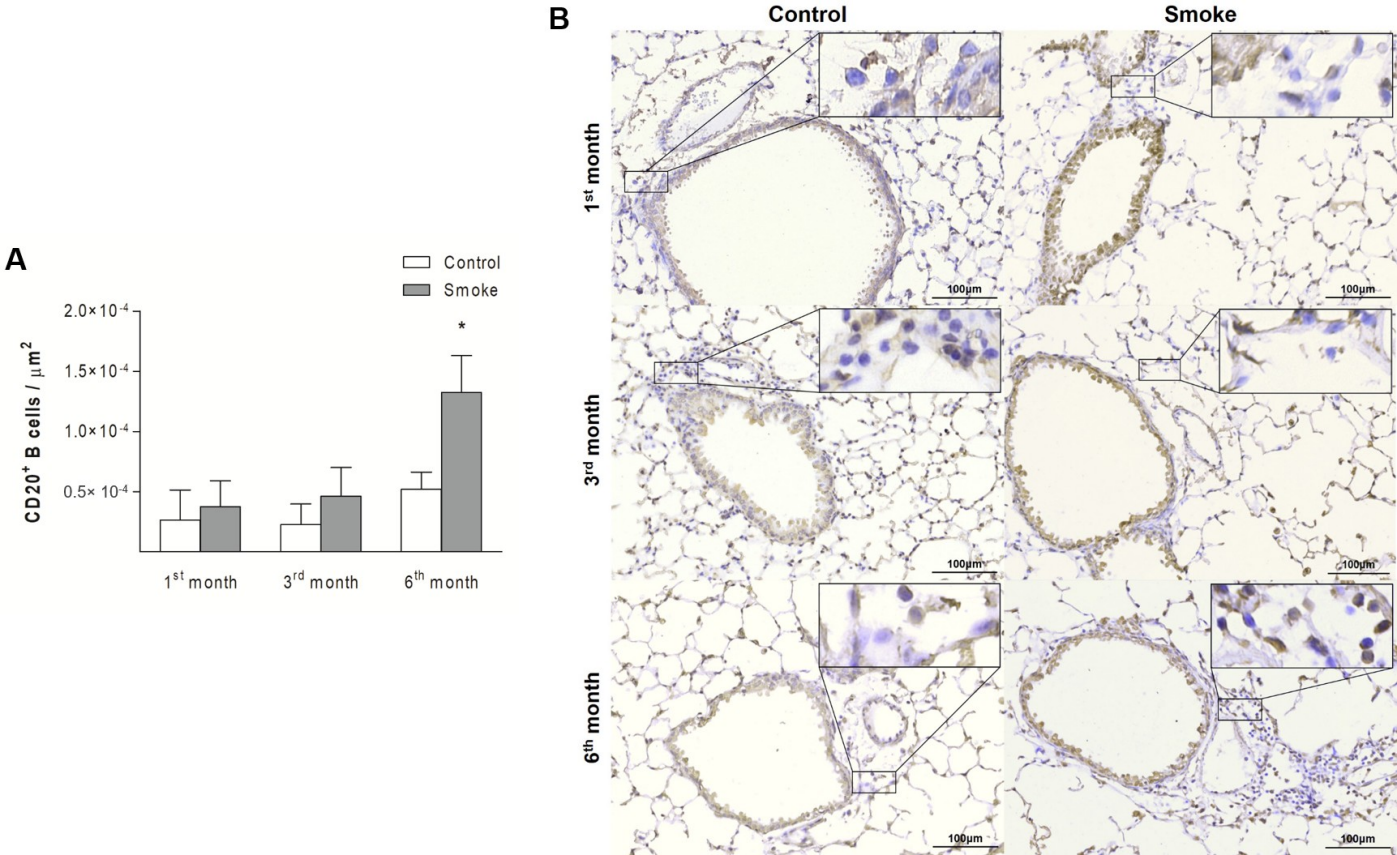
CD20^+^ B cells in peribronchovascular areas. The numbers of CD20^+^ B cells in peribronchovascular areas obtained for the Control groups after 1 (n = 9), 3 (n = 6), and 6 (n = 6) months and the Smoke groups after 1 (n = 9), 3 (n = 7), and 6 (n = 8) months are presented as the means ± SDs. (A) Significant difference was found after 6 (*P = 0.0007, Mann-Whitney test) months. (B) Representative photomicrographs of CD20^+^ B cells in peribronchovascular areas are shown at 200× magnification, and images at 1000× magnification are shown in each insert.

Compared with those in the Control groups, the mice in the Smoke groups showed increases in the numbers of CD4^+^ T cells (Fig 4; 1^st^ month, P = 0.3757, t- test; 3^rd^ month, P = 0.0190, Mann-Whitney test; 6^th^ month, P = 0.0023, Mann-Whitney test) and CD8^+^ T cells (Fig 5; 1^st^ month, P = 0.4359, Mann-Whitney test; 3^rd^ month, P = 0.0002, t-test; 6^th^ month, P = 0.0014, t-test) after exposure for 3 and 6 months. The analysis of CD20^+^ B cells revealed an increase only after 6 months of CS exposure (Fig 6; 1^st^ month, P = 0.3376, t- test; 3^rd^ month, P = 0.0712, t-test; 6^th^ month, P = 0.0007, Mann-Whitney test).

**Fig 7:**
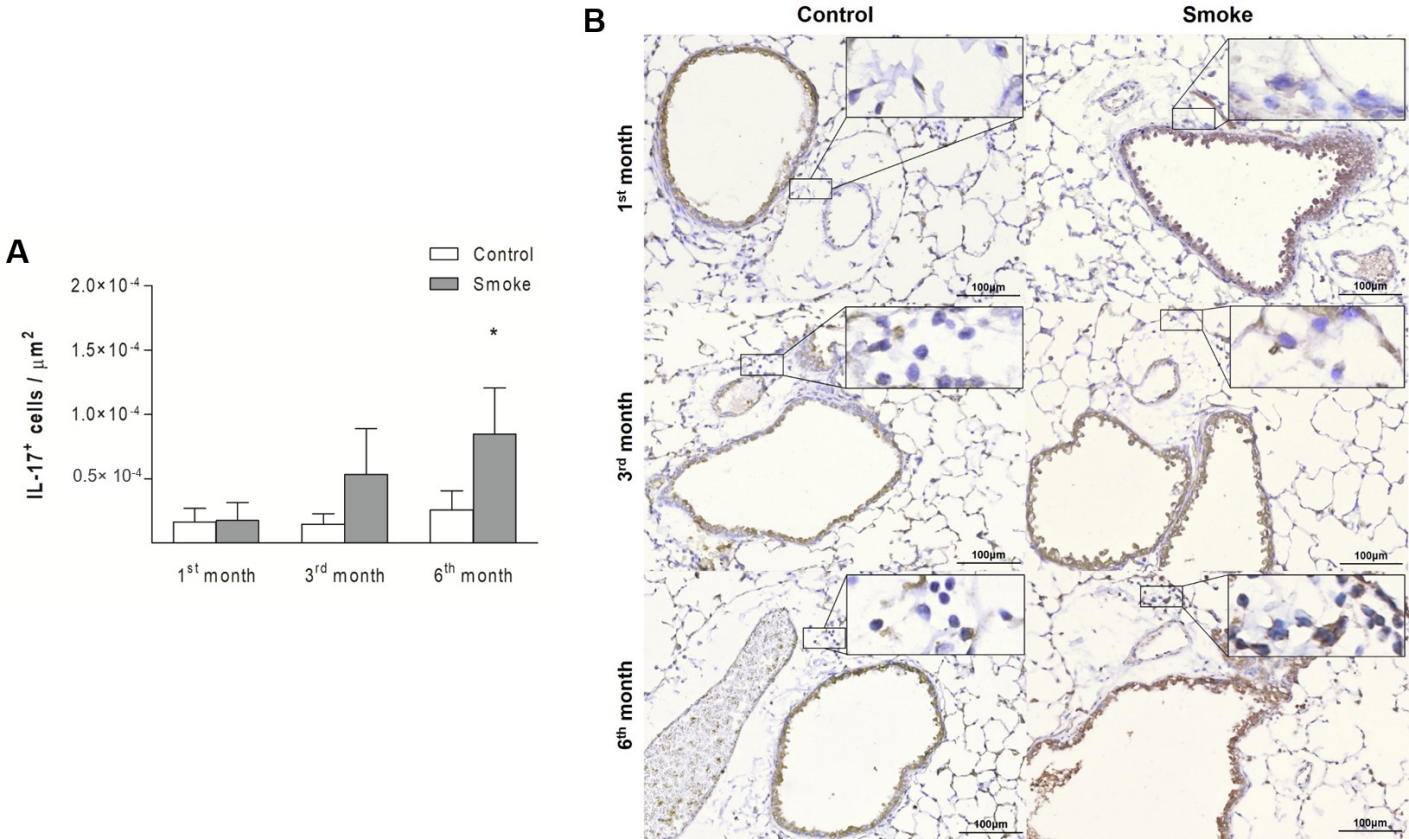
IL-17^+^ cells in peribronchovascular areas. The numbers of IL-17^+^ cells in peribronchovascular areas obtained for the Control groups after 1 (n = 10), 3 (n = 6), and 6 (n = 5) months and Smoke groups after 1 (n = 9), 3 (n = 6), and 6 (n = 9) months are presented as the means ± SDs. (A) Significant difference was found after 6 (*P = 0.0048, t-test) months. (B) Representative photomicrographs of IL-17^+^ cells in peribronchovascular areas are shown at 200× magnification, and images at 1000× magnification are shown in each insert.

**Fig 8:**
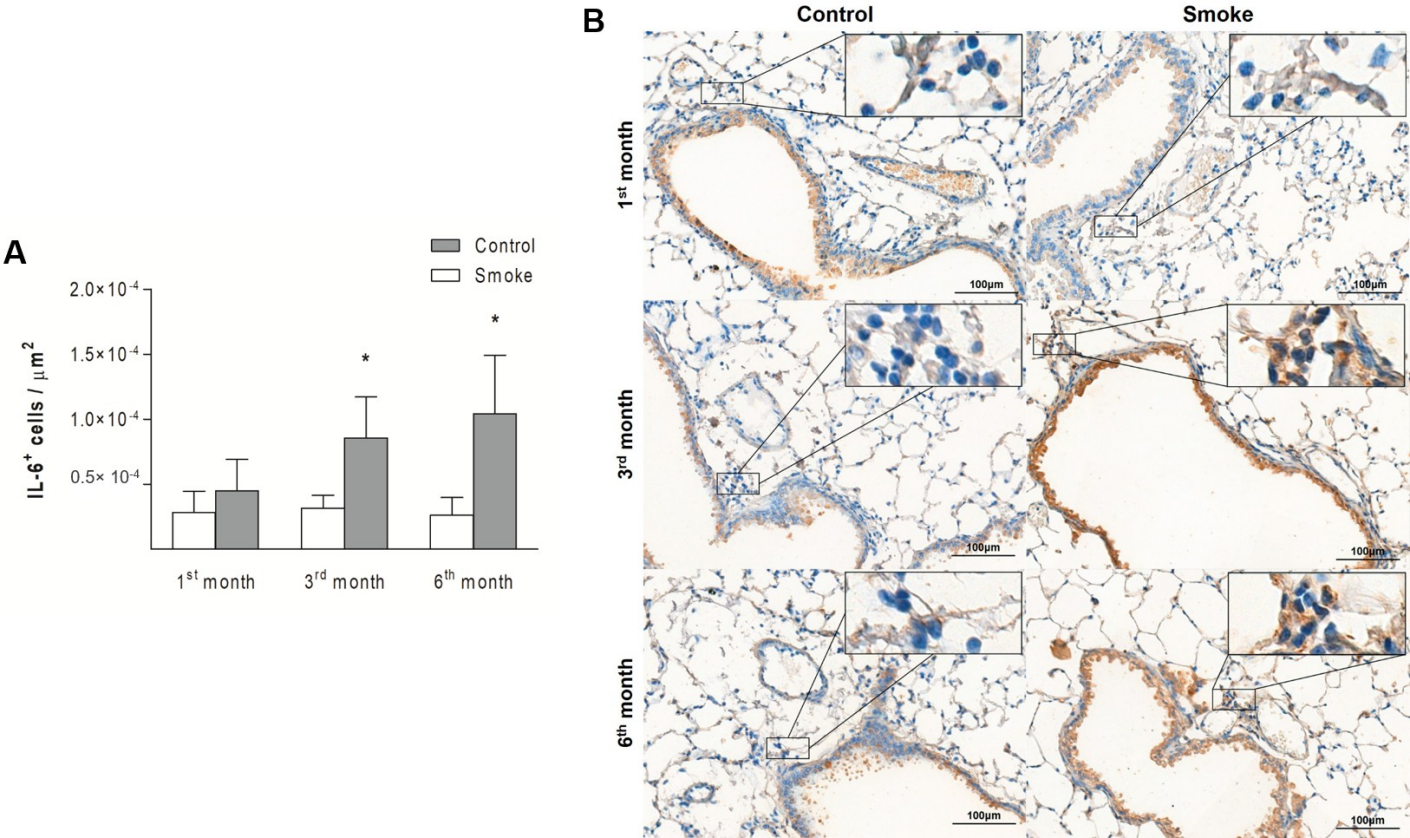
IL-6^+^ cells in peribronchovascular areas. The numbers of IL-6^+^ cells in peribronchovascular areas obtained for the Control groups after 1 (n = 6), 3 (n = 5), and 6 (n = 4) months and Smoke groups after 1 (n = 6), 3 (n = 5), and 6 (n = 6) months are presented as the means ± SDs. (A) Significant differences were found after 3 (*P = 0.0065, t-test) and 6 (*P = 0.0108, t-test) months. (B) Representative photomicrographs of IL-6^+^ cells in peribronchovascular areas are shown at 200× magnification, and images at 1000× magnification are shown in each insert.

An increase in IL-17^+^ cells was detected in the Smoke group compared with the Control group only after 6 months of exposure (Fig 7; 1^st^ month, P = 0.8458, t- test; 3^rd^ month, P = 0.0931, Mann-Whitney test; 6^th^ month, P = 0.0048, t-test). The numbers of IL-6^+^ cells showed an increase after 3 months of CS exposure, and this increase was also observed after 6 months (Fig 8; 1^st^ month, P = 0.2014, t- test; 3^rd^ month, P = 0.0065, t-test; 6^th^ month, P = 0.0108, t-test).

**Fig 9:**
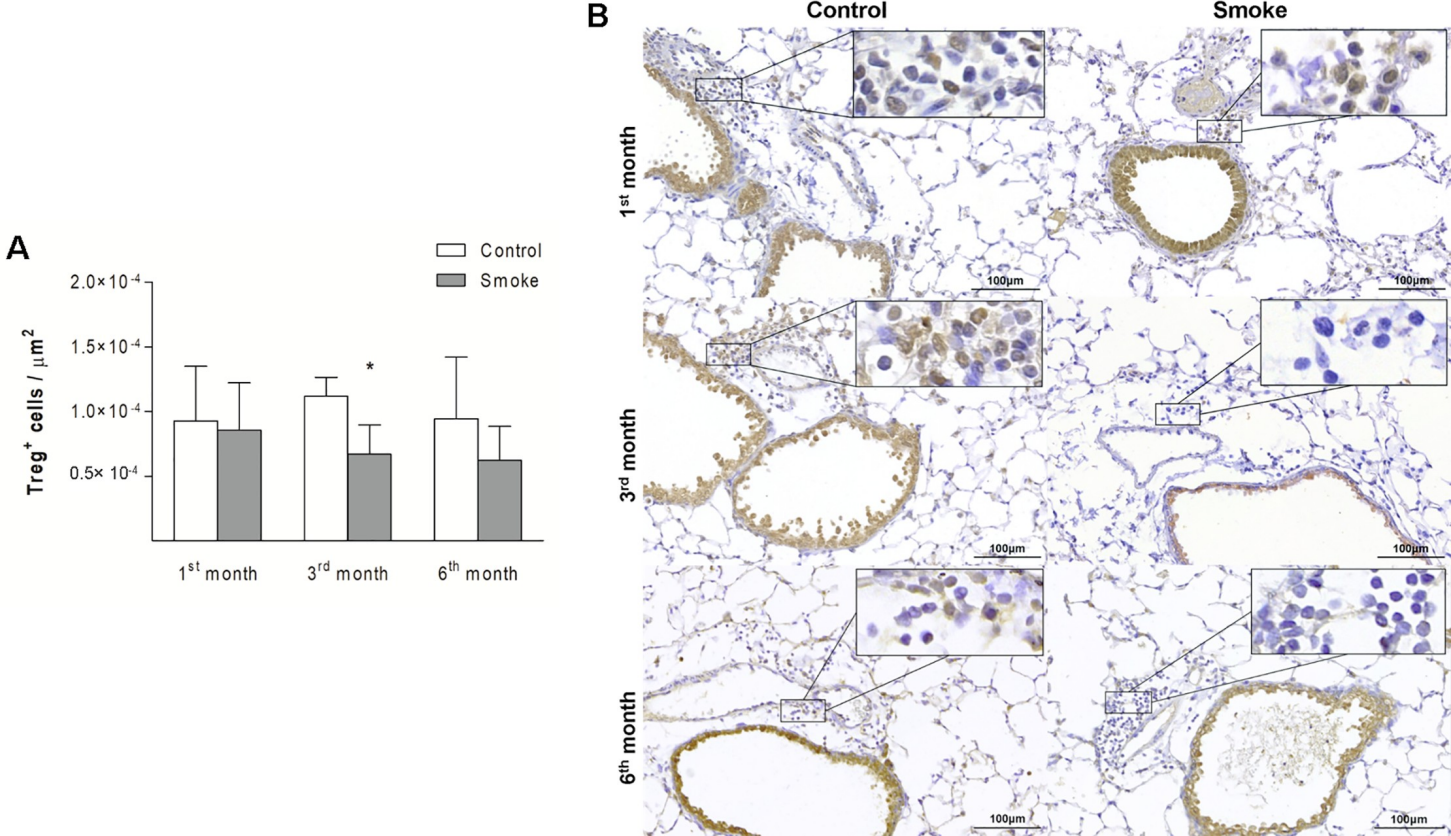
Treg cells in peribronchovascular areas. The numbers of Treg cells in peribronchovascular areas obtained for the Control groups after 1 (n = 8), 3 (n = 6), and 6 (n = 5) months and Smoke groups after 1 (n = 7), 3 (n = 6), and 6 (n = 10) months are presented as the means ± SDs. (A) Significant difference was found after 3 (*P = 0.0024, t-test) months. (B) Representative photomicrographs of Treg cells in peribronchovascular areas are shown at 200× magnification, and images at 1000× magnification are shown in each insert.

**Fig 10:**
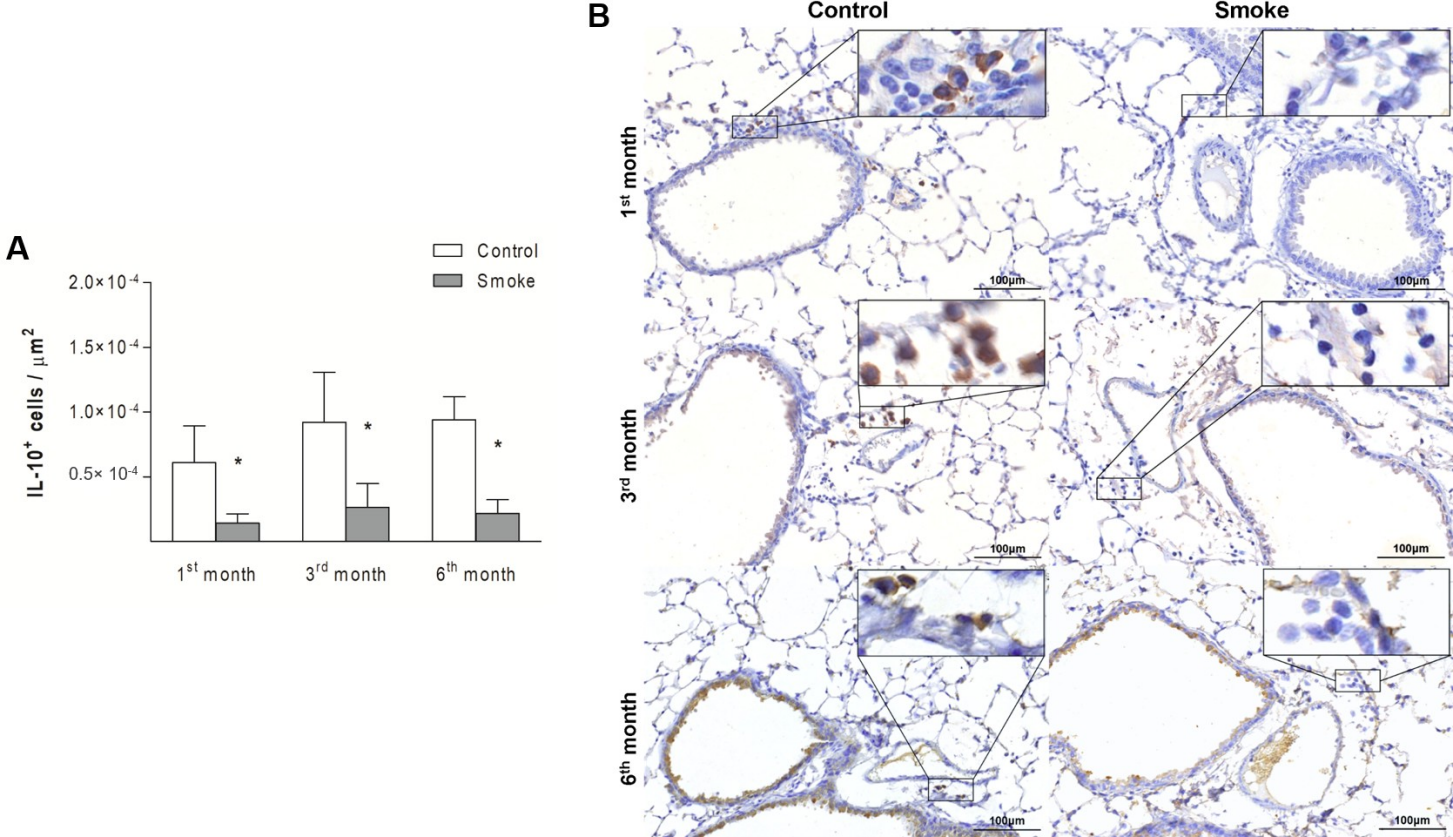
IL-10^+^ cells in peribronchovascular areas. The numbers of IL-10^+^ cells in peribronchovascular areas obtained for the Control groups after 1 (n = 9), 3 (n = 7), and 6 (n = 5) months and Smoke groups after 1 (n = 10), 3 (n = 6), and 6 (n = 9) months are presented as the means ± SDs. (A) Significant differences were found after 1 (*P = 0.0001, Mann-Whitney test), 3 (*P = 0.0029, t-test), and 6 (*P = 0.0001, t-test) months. (B) Representative photomicrographs of IL-10^+^ cells in peribronchovascular areas are shown at 200× magnification, and images at 1000× magnification are shown in each insert.

**Fig 11:**
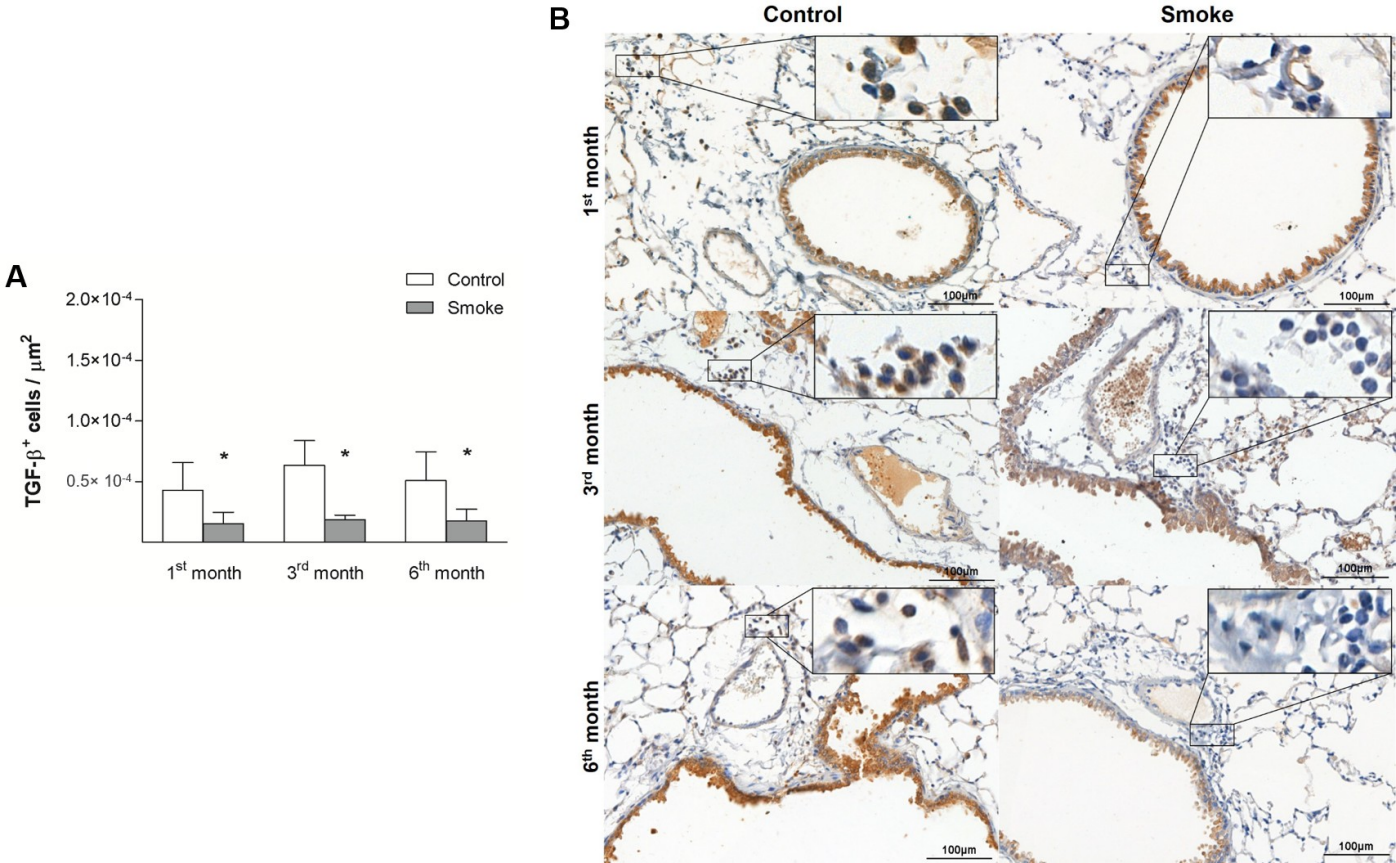
TGF-β^+^ cells in peribronchovascular areas. The numbers of TGF-β^+^ cells in peribronchovascular areas obtained for the Control groups after 1 (n = 4), 3 (n = 4), and 6 (n = 4) months and Smoke groups after 1 (n = 6), 3 (n = 5), and 6 (n = 6) months are presented as the means ± SDs. (A) Significant differences were found after 1 (*P = 0.0472, t-test), 3 (*P = 0.0024, t-test), and 6 (*P = 0.0140, t-test) months. (B) Representative photomicrographs of TGF-β^+^ cells in peribronchovascular areas are shown at 200× magnification, and images at 1000× magnification are shown in each insert.

Compared with the corresponding Control groups, the Smoke groups showed a decrease in the numbers of Treg cells only after 3 months (Fig 9; 1^st^ month, P = 0.9472, t- test; 3^rd^ month, P = 0.0024, t-test; 6^th^ month, P = 0.0535, t-test), whereas significant decreases in IL-10^+^ cells (Fig 10; 1^st^ month, P = 0.0001, Mann-Whitney test; 3^rd^ month, P = 0.0029, t-test; 6^th^ month, P = 0.0001, t-test) and TGF-β^+^ cells (Fig 11; 1^st^ month, P = 0.0472, t- test; 3^rd^ month, P = 0.0024, t-test; 6^th^ month, P = 0.0140, t-test) were observed after 1 month of CS exposure and onward.

### Comparisons among Smoke groups at different time points

**Fig 12:**
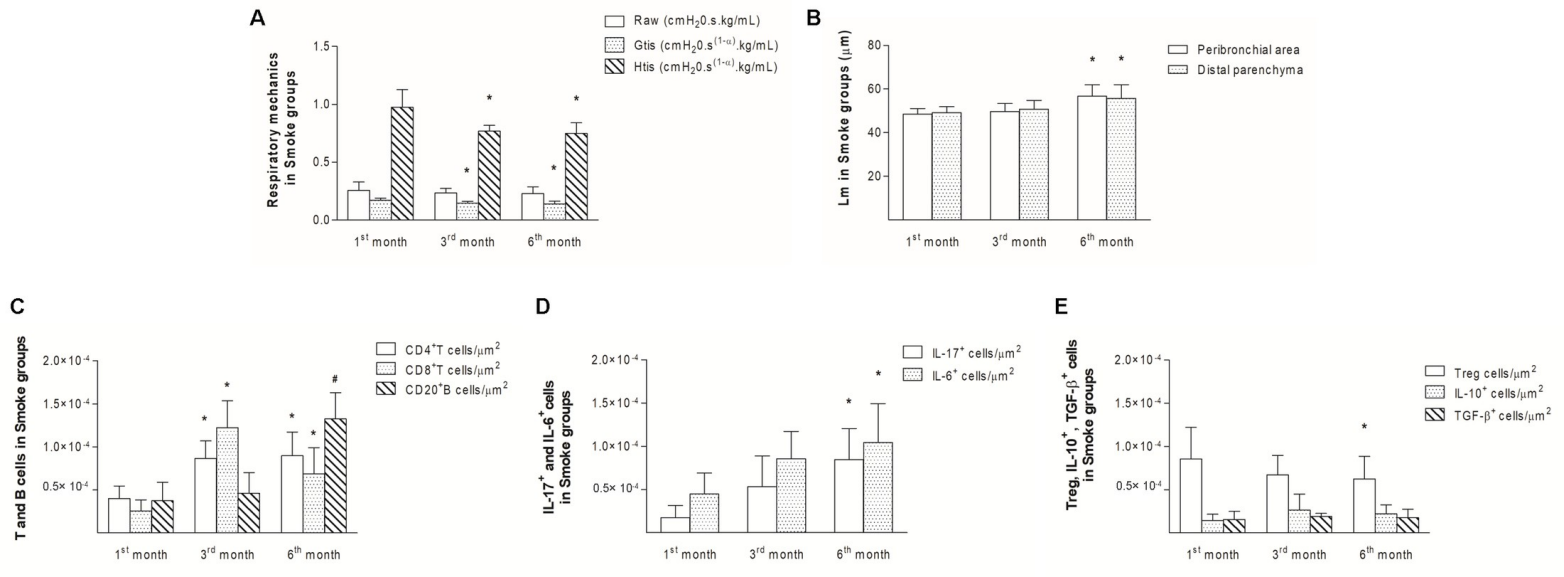
Temporal analysis of the Smoke groups. The respiratory mechanics (A), Lm values (B), T and B cells (C), IL-17^+^ or IL-6^+^ cells (D), Treg, IL-10^+^ or TGF-β^+^ cells (E) in peribronchovascular areas in the Smoke groups after 1, 3, and 6 months are presented as the means ± SDs. (A) The Gtis (*P = 0.0014, One-way ANOVA and Tukey’s post-test) and Htis (*P = 0.0004, One-way ANOVA and Tukey’s post-test) after 3 and 6 months were significantly different from those found after 1 month. (B) The Lm values in the peribronchial area (*P = 0.0019, Kruskal-Wallis test and Dunn’s post-test) and distal parenchyma (*P = 0.0202, Kruskal-Wallis test and Dunn’s post-test) after 6 months were significantly different from those found after 1 month. (C) The numbers of CD4^+^ T cells (*P = 0.0003, One-way ANOVA and Tukey’s post-test) and CD8^+^ T cells (*P < 0.0001, Kruskal-Wallis test and Dunn’s post-test) after 3 and 6 months were significantly different from those found after 1 month. The numbers of CD20^+^ B cells (^#^P < 0.0001, One-way ANOVA and Tukey’s post-test) after 6 months were significantly different from those found after 1 and 3 months. (D) The numbers of IL-17^+^ (*P = 0.0004, One-way ANOVA and Tukey’s post-test) and IL-6^+^ cells (*P = 0.0307, One-way ANOVA and Tukey’s post-test) after 6 months were significantly different from those found after 1 month. (E) The numbers of Treg cells (*P = 0.0004, One-way ANOVA and Tukey’s post-test) after 6 months were significantly different from those found after 1 month.

Compared with the first month, decreases in Gtis and Htis were observed after 3 months of exposure and onward (Fig 12A; Raw, P = 0.3273, One-way ANOVA and Tukey’s post-test; Gtis, P = 0.0014, One-way ANOVA and Tukey’s post-test; Htis, P = 0.0004, One-way ANOVA and Tukey’s post-test). Additionally, the Lm values were increased after 6 months in both the peribronchial area and distal parenchyma compared with the same areas after one month (Fig 12B; Peribronchial area, P = 0.0019, Kruskal-Wallis test and Dunn’s post-test; Distal parenchyma, P = 0.0202, Kruskal-Wallis test and Dunn’s post-test), which suggested progressive functional and structural changes.

The analysis of the immune response revealed increases in CD4^+^ and CD8^+^ T cells after 3 months compared with after one month, and increases in these cells were still observed after 6 months. The numbers of CD20^+^ B cells were increased after 6 months compared with the numbers observed after 1 and 3 months (Fig 12C; CD4^+^ T cells, P = 0.0003, One-way ANOVA and Tukey’s post-test; CD8^+^ T cells, P < 0.0001, Kruskal-Wallis test and Dunn’s post-test; CD20^+^ B cells, P < 0.0001, One-way ANOVA and Tukey’s post-test).

After 6 months, we observed an imbalance between the Th17/Treg cells, which was represented by significant increases in IL-17^+^ cells and IL-6^+^ cells (Fig 12D; IL-17^+^ cells, P = 0.0004, One-way ANOVA and Tukey’s post-test; IL-6^+^ cells, P = 0.0307, One-way ANOVA and Tukey’s post-test) and a concomitant decrease in Treg cells (Fig 12E; Treg cells, P = 0.0004, One-way ANOVA and Tukey’s post-test) relative to the numbers observed after 1 month. No differences in IL-10^+^ or TGF-β^+^ cells were observed (Fig 12E; IL-10^+^ cells, P = 0.2069, Kruskal-Wallis test and Dunn’s post-test; TGF-β^+^ cells, P = 0.2048, One-way ANOVA and Tukey’s post-test).

### Immunofluorescence analysis

**Fig 13:**
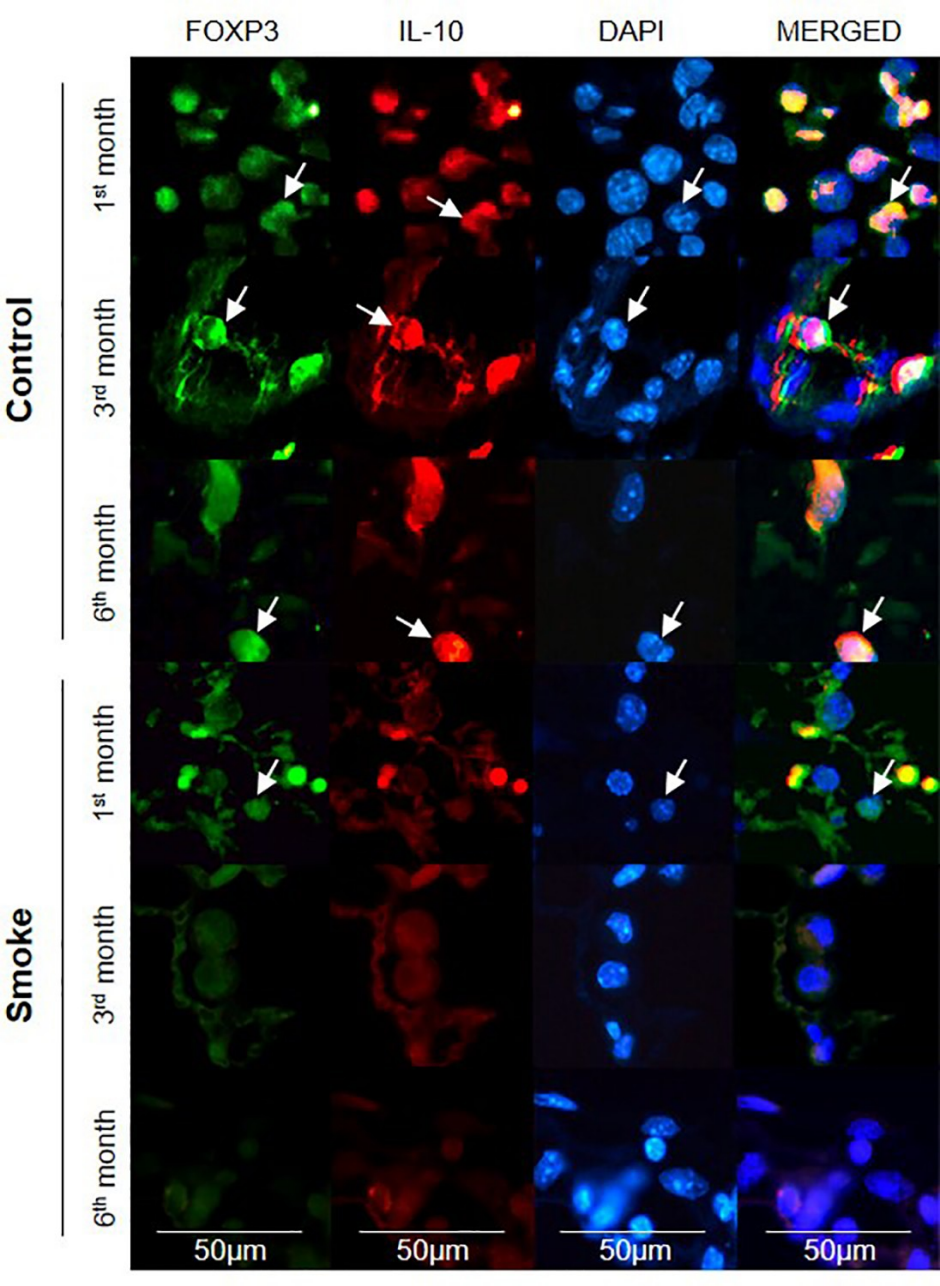
Representative immunofluorescent images showing FOXP3 and IL-10 staining. The expression of FOXP3 (green) and IL-10 (red) in lymphocytes that infiltrated the peribronchovascular areas of the Control and Smoke groups after 1, 3, and 6 months was assessed by double-label immunofluorescence. The nuclei were labeled with DAPI (blue), and the arrows indicate positive cells. The images are shown at 400× magnification.

The results from immunofluorescence analysis was consistent with the immunohistochemical findings. The merged images revealed that the FOXP3^+^ cells (green) colocalized with the IL-10^+^ cells (red) and nuclei (blue) in all the control groups. The analysis of the Smoke groups after 1 month revealed only a few IL-10^+^ cells (red), whereas exposure to CS for 3 and 6 months resulted in weak signals for both FOXP3^+^ and IL-10^+^ cells (Fig 13).

## Discussion

Our results show that the microenvironmental stimuli produced by pro- and anti-inflammatory cytokines during COPD progression lead to a Th17/Treg imbalance. To our knowledge, this study constitutes the first evaluation of Th17 and Treg cells and some of their specific cytokines at different time points during COPD development.

An increase in IL-17^+^ cells and a decrease in Treg cells were detected after six months compared with after one month, and at this time period we also observed higher Lm values in the peribronchial area and the distal parenchyma, which suggested a progression of alveolar enlargement over time. This progression of alveolar enlargement and inflammatory processes at different time points agreed with the respiratory mechanics findings.

We observed a transitory increase in tissue elastance after one month of CS exposure, and this increase was likely due to the presence of tissue edema and inflammatory infiltrates in the lung parenchyma [28]. However, from three months to the end of the exposure protocol, we detected a worsening of lung function, characterized by decreases in tissue elastance and tissue resistance. This finding corroborates previous studies that highlighted the pivotal role of a Th17/Treg imbalance in lung function impairment [11, 16, 29].

Decreases in IL-10^+^ cells and TGF-β^+^ cells occurred before the reduction in Treg cells, suggesting that despite the relative abundance of Treg cells after one month of CS exposure, these cells likely exhibit reduced immunosuppressive activity (Fig 13). Such results corroborate previous findings that note the critical role of TGF-β in inducing the differentiation of Tregs with immunosuppressive activity [19, 30].

Previous clinical findings have shown the importance of Treg cells and IL-10 production in obstruction progression in smokers [11, 13, 14, 29]. Sales et al. [14] reported decreases in Treg cells and IL-10^+^ cells and a consequent increase in IL-17^+^ cells in the airways of obstructed smokers compared with those of healthy smokers and control subjects [14]. Additionally, Wang et al. [11] reported decreases in the frequency of Tregs and the levels of IL-10 in the peripheral blood and serum, respectively, in patients with moderate and severe COPD compared with heavy smokers and healthy controls [11].

Through an experimental comparison of CS-exposed animals with air-exposed controls, Wang et al. [15] found initial increases in Treg cells and IL-10 expression in the first month and decreases in the sixth month [15]. Duan et al. [16] also showed reductions in Treg cells and IL-10 expression with a concomitant increase in IL-17 in the third month of CS exposure, and these changes were intensified in the sixth month [16].

The balance between Th17 and Treg cells is essential for immune homeostasis [19, 29]. In our study, the Th17 response was observed in later stages after an increase in IL-6^+^ cells, and this finding reinforces the relevance of this cytokine for the differentiation of Th17 cells and the inhibition of Treg differentiation [11, 29].

The potential inflammatory property of Th17 cells is mainly mediated by IL-17, which has been associated with the development of inflammatory lung diseases, such as COPD [31–33]. Chen et al. [34] reported that IL-17^-/-^ mice fail to develop emphysema even after 6 months of CS exposure, reinforcing the pathogenic role of this cytokine [34]. Additionally, Kurimoto et al. [35] observed reductions in neutrophil recruitment and emphysematous changes in the lungs of IL-17^-/-^ mice subjected to elastase instillation [35].

Furthermore, this cytokine is important in COPD exacerbations [36]. Because treatment with a neutralizing anti-IL-17 antibody attenuates neutrophilic inflammation in mice during COPD exacerbation caused by an infection with nontypeable *Haemophilus influenzae* (NTHi) [37], the targeting of IL-17 constitutes a promising therapeutic strategy for reducing the severity of COPD exacerbation and preventing COPD progression.

Our analyses of CD4^+^ T cells, CD8^+^ T cells and B cells revealed increases in the numbers of these cells, as previously observed in COPD patients [8, 9, 14, 17, 38] and animal models [15, 16, 39, 40]. Specifically, increases in CD4^+^ T cells and CD8^+^ T cells were observed after three months of CS exposure and continued to be observed after six months. In contrast, the B cell numbers were increased only at the end of the protocol.

Increases in B cells have been observed in the small [8] and large [41] airways, and were found to be associated with COPD progression. Moreover, these cell types organize into lymphoid follicles around the small airways and in the lung parenchyma of COPD patients [8, 39]. Although we observed increased numbers of B cells in the lung parenchyma, we did not detect lymphoid follicles in this CS-induced model at the various time points.

Considering the importance of the innate immune response in COPD development and the role of the epithelium in this response [6, 42], we evaluated the NF-kB and TNF-α expression levels in peribronchovascular areas and the respiratory epithelium isolated by laser microdissection. We obtained similar results for both compartments: an increase in NF-κB^+^ cells was observed after one month of CS exposure and onward, and increases in TNF-α^+^ cells and the gene expression of both inflammatory mediators were detected after three months and onward.

The comparison of our results with previous studies using human lung samples [10, 14] revealed that the main difference is the lung compartments in which we detected the inflammatory signal: in humans, the inflammatory infiltrate has mainly been observed in the airways between the respiratory epithelium and adventitia [8, 14], whereas in this animal model, we observed the inflammatory infiltrates mostly in peribronchovascular areas between the airways and adjacent blood vessels. This difference likely occurs due to the specialized capillaries in this area, which allow the rapid release of leukocytes and thereby results in edema and inflammatory infiltration [43].

Although the pathophysiological features of COPD show many differences between humans and animal models, it is important to note that we observed the same inflammatory profile, with increases in T and B cells and a reduction in Treg cells, in both organisms.

## Conclusion

Our results show that the Th17/Treg imbalance plays a pivotal role in COPD progression and reinforce the importance of the microenvironmental stimuli produced by the released pro- and anti-inflammatory cytokines in this response.
